# Metatranscriptomic shifts suggest shared biodegradation pathways for Corexit 9500 components and crude oil in Arctic seawater

**DOI:** 10.1111/1758-2229.13127

**Published:** 2022-09-29

**Authors:** Taylor R. Gofstein, Mary Beth Leigh

**Affiliations:** ^1^ Institute of Arctic Biology University of Alaska Fairbanks Fairbanks Alaska USA; ^2^ Department of Chemistry & Biochemistry University of Alaska Fairbanks Fairbanks Alaska USA; ^3^ Department of Biology and Wildlife University of Alaska Fairbanks Fairbanks Alaska USA

## Abstract

While the genes and pathways responsible for petroleum biodegradation in marine environments have received substantial attention, considerably less is known about those active in the biodegradation of the commonly applied chemical dispersant Corexit 9500. Yet, their fate in the Arctic marine environment is an increasingly important unknown. To elucidate the genes and pathways active in the biodegradation of oil and dispersants, we performed metatranscriptomic sequencing on microbial communities in Arctic seawater exposed to oil, Corexit, or both for 0, 5, and 30 days in a mesocosm incubation experiment. While oil and Corexit stimulated significantly different metatranscriptomic profiles overall, both enriched a suite of fatty acid degradation gene transcripts. Based on the gene transcripts observed and the chemical structures of Corexit 9500 surfactant components, we propose a hypothetical pathway for Corexit surfactant biodegradation in which surfactant ester groups are transformed into fatty acids that are then funnelled into the β‐oxidation fatty acid degradation pathway. Several microbial taxa within Oceanospirillales, Pseudomonadales, and Alteromonadales were associated with either oil‐only or Corexit‐only exposure, potentially implicating them in the degradation of these mixtures. Metabolic gene transcripts were associated with diverse gammaproteobacterial lineages, with many genera exhibiting functional redundancy. These findings offer new insight into the potential genes, pathways, and microbial consortia involved in the biodegradation of Corexit 9500 in the Arctic marine environment.

## INTRODUCTION

Chemical dispersants such as Corexit 9500 are frequently applied to oil spills to promote the formation of small oil droplets that can be dispersed throughout the water column, thereby reducing the hazards associated with a surface oil slick. This droplet formation also increases bioavailability of oil to indigenous microorganisms and may help increase access to nutrients for microbes as oil droplets disperse through the water column. The majority of studies of Corexit 9500 report increased oil biodegradation in seawater when compared to non‐dispersed slicks (Prince & Butler, 2014) or to physically dispersed oil (Brakstad et al., 2014, 2018; McFarlin et al., 2014; Prince et al., 2013), including in Arctic seawater, although the inhibition of oil‐degrading bacteria by Corexit has been reported in some studies in temperate regions (Kleindienst et al., 2015; Rahsepar et al., 2016). While the biodegradation of petroleum hydrocarbons, including the genes and pathways active under a variety of environmental conditions, has been well studied (Das & Chandran, 2011; Harayama et al., 1999), far less is known about the biodegradation of the chemical components of Corexit 9500 itself.

Corexit 9500 is largely composed of the anionic surfactant DOSS and the non‐ionic surfactants Span 80, Tween 80, and Tween 85 in a solvent composed of hydrocarbon distillates. Following the 2010 Deepwater Horizon oil spill and subsequent application of Corexit 9500, in situ measurements showed a persistence of DOSS (Kujawinski et al., 2011; Perkins et al., 2017; White et al., 2014), indicating the recalcitrance of at least one component of Corexit. Recently, the biodegradation of DOSS and Corexit's nonionic surfactant components have been observed when Corexit was incubated in Arctic seawater mesocosms alone and in the presence of oil (Gofstein et al., 2020; McFarlin et al., 2018). A Geochip assay of Arctic seawater exposed to either oil or Corexit separately showed increased intensities of hydrocarbon degradation genes for both treatments relative to controls (McFarlin et al., 2018), suggesting that Corexit biodegradation may share some genes or pathways with oil biodegradation.

In this study, we sought to increase our understanding of the microbial biodegradation of Corexit 9500 and chemically dispersed crude oil in Arctic seawater by addressing the following aims: (1) identify metabolic genes associated with Corexit and oil biodegradation by comparing those expressed during their active biodegradation, both alone and in combination; (2) construct a putative pathway for the biodegradation of Corexit's surfactant components; and (3) elucidate the roles of different members of the oil‐ and dispersant‐degrading microbial consortium in Arctic seawater. We hypothesized that some microorganisms are capable of biodegrading components of both oil and Corexit and utilize some of the same alkane hydrocarbon‐degradation genes and pathways to do so.

## EXPERIMENTAL PROCEDURES

Samples used for this study were obtained from a previously performed mesocosm experiment as described in Gofstein et al., 2020. Mesocosms containing 800 ml of seawater collected from the Chukchi Sea were treated with either 50 ppm Alaska North Slope crude oil, 5 ppm Corexit 9500, both (1:20 dispersant‐to‐oil ratio), or neither (unamended control) and incubated on stir plates at 4°C on a 19‐h day/5‐h night light cycle for 30 days. Mesocosms for all treatment and time point combinations were prepared in triplicate (*n* = 3). The 0‐, 5‐, and 30‐day time points were selected based on prior knowledge and predictions to capture the active degradation of Corexit 9500 in Arctic conditions. At each specified time point, samples for microbial analysis were collected by vacuum filtration onto 0.22‐μm filters, which were then immediately frozen at −80°C until extraction of RNA. Prior analyses included quantitation of petroleum hydrocarbon losses using gas chromatography–mass spectrometry (GC/MS), Corexit 9500 surfactant component (DOSS, Span 80, Tweens 80 and 85) losses using liquid chromatography tandem mass spectrometry (LC/MS/MS), nutrient analyses, and 16S rRNA gene amplicon sequencing (Gofstein et al., 2020).

RNA was extracted from filters using a Qiagen RNeasy Lipid tissue kit following the manufacturer's protocol; this kit was selected to help ensure high extraction yields in the presence of oil. RNA extracts were held at −80°C and shipped on dry ice to the Oregon State University Center for Genome Research and Biocomputing where they were quantified on an Agilent Bioanalyzer 2100, eukaryote ribodepleted with the Ribo‐Zero rRNA Removal Library Prep kit, prepared for sequencing using the TruSeq RNA sample preparation protocol, pooled, and sequenced on an Illumina HiSeq 3000 using a 100‐bp paired‐end format. Sequences were processed using the MG‐RAST pipeline (v.4.0.3), analysed against the RefSeq phylogenetic and Kyoto Encyclopedia of Genes and Genomes (KEGG) functional databases (Glass & Meyer, 2011; Kanehisa et al., 2016; O'Leary et al., 2016), and normalized to the total number of sequence counts per sample. All rRNA sequences were removed from the dataset during processing to focus on metabolic mRNA transcripts. Sequencing efforts generated 2,092,023,528 sequences prior to data processing that passed quality‐control filters across 27 individual samples for an average of 76,340,561 sequences per sample. Statistical analyses were performed using the PC‐ORD V6 statistical software package (PC‐ORD v. 6.255 Beta. Gleneden Beach, OR: MjM Software Design; McCune et al., 2002) and the DESeq2 R Bioconductor package (v. 3.10). Analyses included non‐metric multidimensional scaling (NMDS) plots to visualize metatranscriptomes, permutational multivariate ANOVA (perMANOVA) tests to evaluate the statistical significance of observed differences, Mantel tests to correlate the abundance of metabolic gene classes with the overall metatranscriptomes, and Wald tests with Benjamini‐Hochberg adjusted *p*‐values to determine if relative expression of gene transcripts differed between treatments at a given timepoint. All statistical tests were performed using a 95% confidence interval.

## RESULTS

Samples used in this study were obtained from an experiment in which Arctic seawater was incubated with crude oil, Corexit 9500 or both together, as described previously (Gofstein et al., 2020). Previous analyses referenced here include quantitation of oil loss, Corexit 9500 surfactants, nutrient analyses, and 16S rRNA gene sequencing. For this study, we subjected samples collected from the 0‐, 5‐, and 30‐day time points of the incubation experiment to metatranscriptomics analyses. These time points were selected based on the findings of the previous chemical analyses demonstrating active biodegradation.

### Overall metabolic metatranscriptome

**FIGURE 1:**
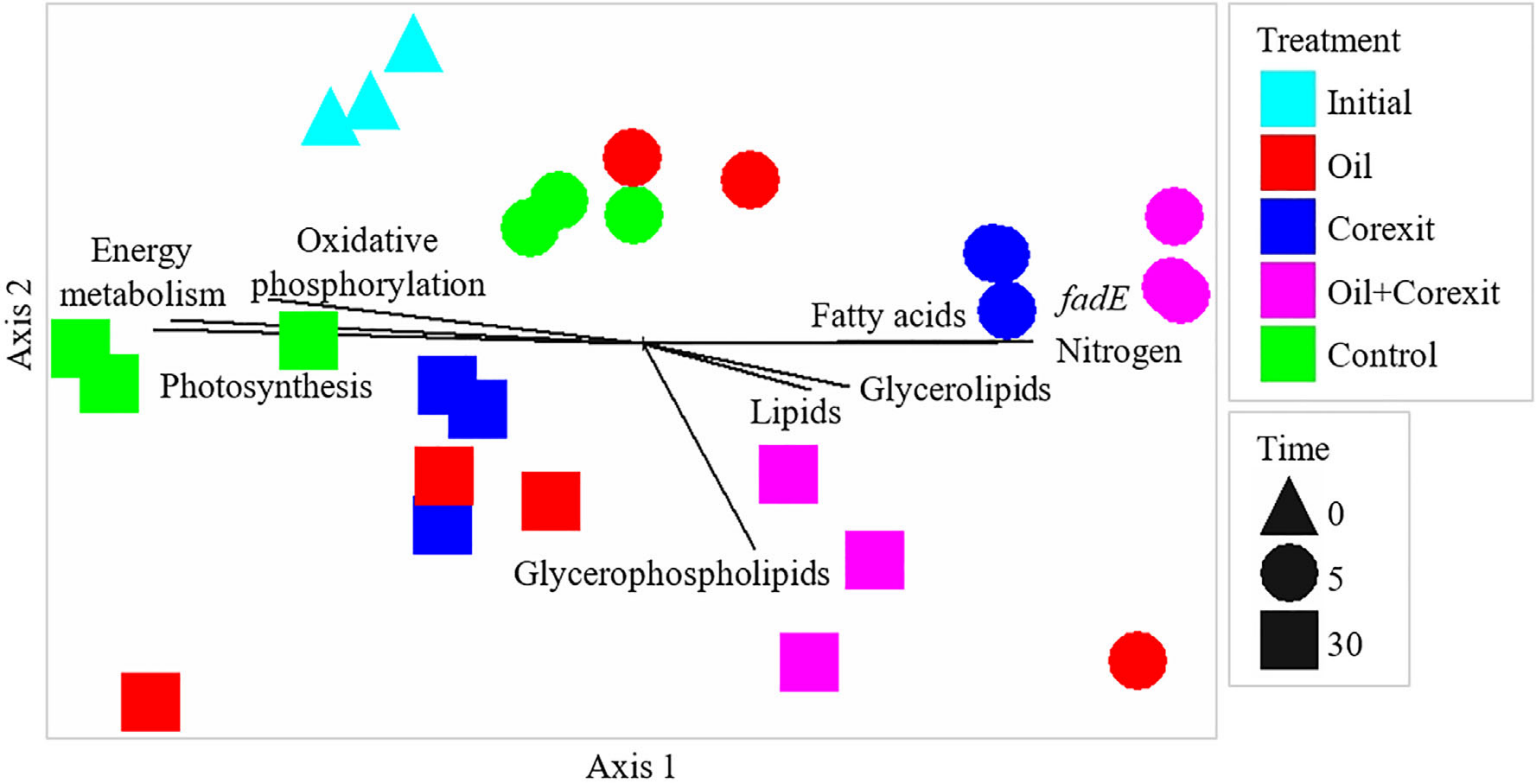
Bray‐Curtis transformed NMDS of Arctic seawater metabolic gene metatranscriptomes at 0, 5, and 30 days in mesocosms amended with Corexit and/or oil or no treatment (control). A perMANOVA test was performed for the effects of the treatments applied (*p* = 0.0002), time (*p* = 0.0002), and the interaction of both factors (*p* = 0.0022), with all pair‐wise comparisons between different treatments significant (*p* < 0.02)

Visualization of the metabolic metatranscriptomes with a non‐metric multidimensional scaling (NMDS) plot and a permutational multivariate ANOVA (perMANOVA) test showed significant grouping by the treatments applied (*p* = 0.0002), time (*p* = 0.002), and the interaction of both factors (*p* = 0.0022; Figure 1), with all pair‐wise comparisons significant (*p* < 0.02). The relative abundance of functional RNA transcripts originating from bacteria compared to other microorganisms (archaea, eukaryotes, viruses) was significantly different between treatments, with bacterial transcripts being more enriched (*p* = 0.0145) in treatments containing Corexit compared to oil‐only and the control at day 5 (Figure S1). Transcripts encoding RNA polymerase B (rpoB) were also significantly more enriched in Corexit and oil + Corexit treatments than oil‐only or the control at day 5 (*p* < 0.0001; Figure S2), further suggesting that bacterial gene expression was significantly enriched for treatments containing Corexit early in the incubation relative to the oil‐only treatment.

**FIGURE 2:**
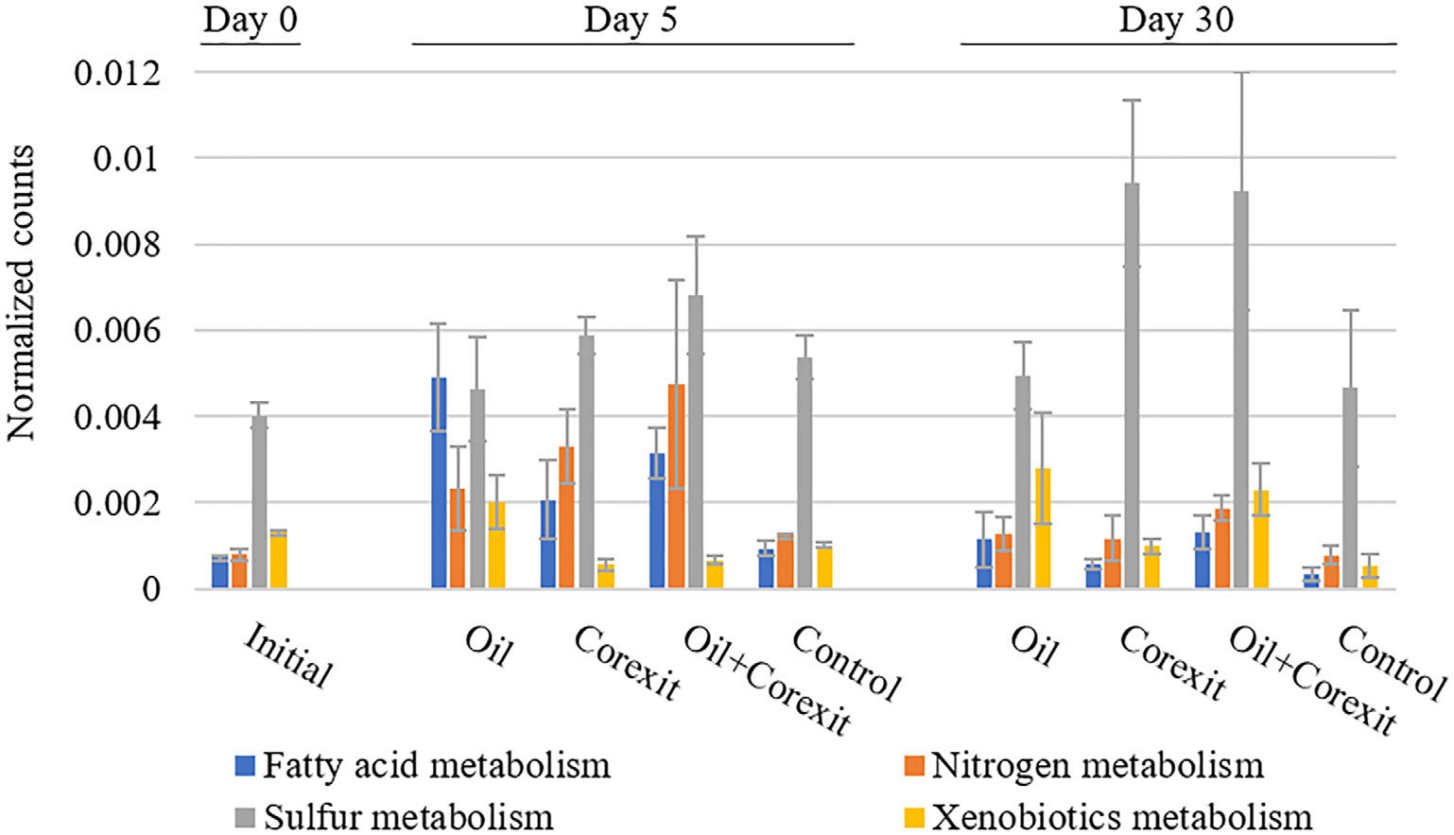
Normalized counts of select metabolic transcripts by treatment at 0, 5, and 30 days in Arctic seawater mesocosms amended with Corexit and/or oil or no treatment (control)

Several classes of metabolic transcripts were enriched in treatments containing Corexit on day 5 relative to the oil treatment and unamended control, including those associated with respiration and the metabolism of nitrogen and sulphur (Figure 2; Supporting Information). A vector analysis of different metabolic gene classes relative to the functional metatranscriptome of each sample (Figure 1) revealed strong correlations (Mantel correlation coefficient, *r* > 0.70) of energy‐metabolism‐related genes with differences in the overall microbial metatranscriptomic structure (Table S1). Moderate correlations of oxidative phosphorylation and nitrogen metabolism genes to metatranscriptomic structure were also detected using vector analysis (0.45 < *r* < 0.70) (Table S1), with weak correlations (*r* < 0.45) associated with lipid, fatty acid, glycerolipid, and glycerophospholipid metabolism genes.

### Enrichment of fatty acid degradation genes

**FIGURE 3:**
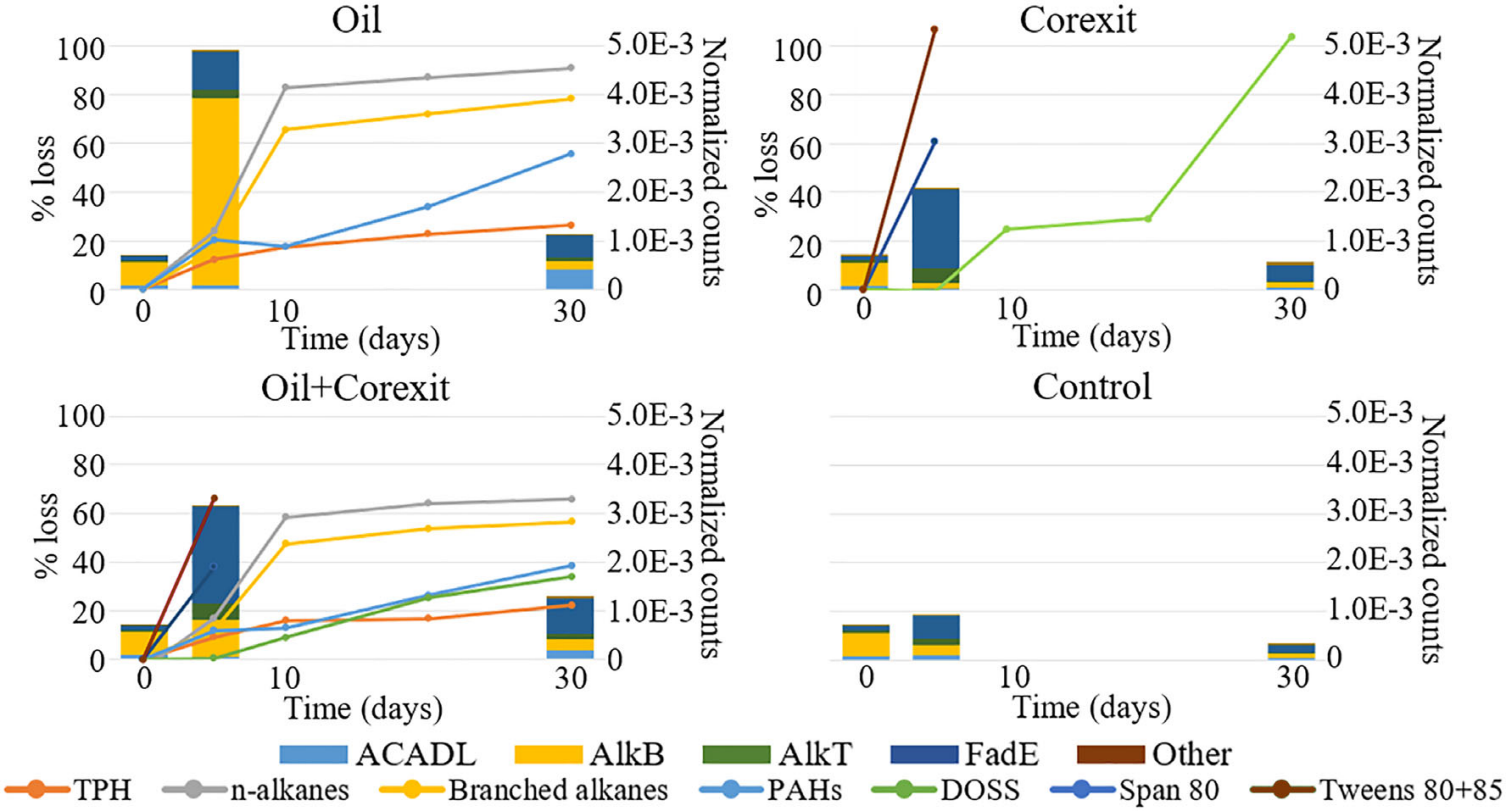
Normalized counts of fatty acid degradation gene transcripts (bars) and the extent of chemical loss (lines; previously reported in Gofstein et al. (2020) at 0, 5, and 30 days for (A) oil only, (B) Corexit only, (C) oil and Corexit, and (D) the unamended control in Arctic seawater mesocosms. The legend abbreviations are as follows: Long chain acyl co‐A dehydrogenase (ACADL), alkane 1‐monooxygenase (alkB), rubredoxin‐NAD+ reductase (AlkT), acyl co‐A dehydrogenase (fadE), total petroleum hydrocarbons (TPH), polycyclic aromatic hydrocarbons (PAHs), dioctyl sodium sulfosuccinate (DOSS)

The presence of oil was associated with significant enrichment of transcripts encoding fatty acid (*p* < 0.05) and xenobiotic (*p* < 0.0001) metabolism genes relative to unamended controls or incubations containing Corexit (Figure 2). Of the fatty acid metabolism genes, those specifically associated with fatty acid degradation showed significant enrichment in oil and oil + Corexit treatments compared to controls at 5 days (*p* = 0.0314; Figure 3). In addition to differences in total fatty acid degradation transcription, different genes within the fatty acid metabolism group appear to have been upregulated in oil versus Corexit treatments. Alkane 1‐monooxygenase (*alkB*), a key enzyme in the degradation of aliphatic hydrocarbons, was significantly more enriched in treatments containing oil (*p* < 0.0001) relative to the other treatments at 5 days (Figure 3) In contrast, transcripts encoding for acyl‐CoA dehydrogenase (*fadE*), which catalyses the first step of fatty acid β‐oxidation, were enriched in treatments containing Corexit compared to oil‐only and the control at 5 days (Figure 3, *p* = 0.0077). Additionally, the subsequent β‐oxidation gene transcripts for acetyl‐CoA acetyltransferase (*fadA*) and 3‐hydroxyacyl‐CoA dehydrogenase (*fadB*) were significantly enriched (*p* = 0.0049 and *p* < 0.0001) in treatments containing Corexit than other treatments at day 5. At day 5, the enrichment of all these β‐oxidation gene transcripts was associated with a significant loss of Corexit non‐ionic surfactant components to below detection limits (Figure 3; Gofstein et al., 2020). However, the abundance of these β‐oxidation genes did not differ between treatments at 30 days, likely due to the extensive loss of Corexit compounds by this timepoint (Figure 3; Gofstein et al., 2020).

### Taxonomic affiliation of transcripts

**FIGURE 4:**
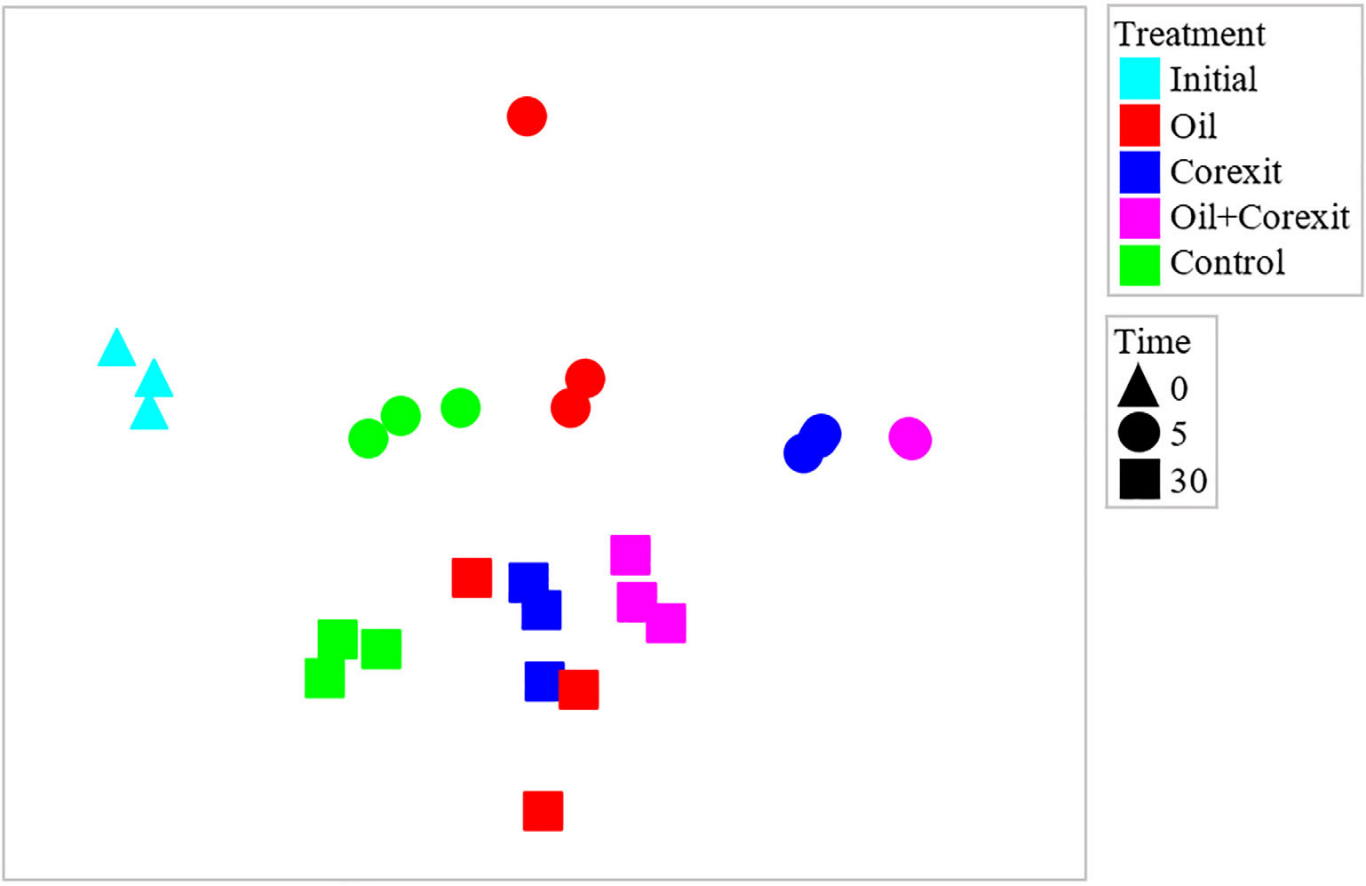
Bray‐Curtis transformed NMDS plot of the phylogenetic affiliation of RNA transcripts in Arctic seawater mesocosms amended with Corexit and/or oil or no treatment (control) at 0, 5, and 30 days incubation. A perMANOVA test was performed for the effects of the treatments applied (*p* = 0.0002), time (*p* = 0.0002), and the interaction of both factors (*p* = 0.0002), with all pair‐wise comparisons between different treatments significant (*p* < 0.05)

The taxa previously reported to proliferate in response to oil and/or Corexit in this incubation study (Gofstein et al., 2020) were associated with enriched gene transcripts for several key metabolic and biogeochemical processes relevant to contaminant degradation including fatty acid degradation, nitrogen cycling, and sulphur cycling (Table 1). Based on the taxonomic affiliations of the metabolic transcripts, different populations became metabolically active in response to oil and/or Corexit (Figure 4). A perMANOVA test showed there were significant effects of treatment (*p* = 0.0002), time (*p* = 0.0002), and the interaction of both (*p* = 0.0002) on bacterial community composition as represented by the origins of metabolic transcripts, with all pair‐wise comparisons showing significant differences (*p* < 0.05). This agrees with prior 16S‐rRNA gene amplicon sequence analyses from this incubation experiment that showed differences between oil and Corexit treatments in overall microbial community structure and the relative abundance of specific taxa, with oil + Corexit incubations resulting in communities representing a mixture of the distinct communities that emerged in response to oil‐only and Corexit‐only treatments.

**TABLE 1:** Summary of genera associated with the expression of genes or metabolic pathway transcripts in Arctic seawater mesocosms

Gene or metabolic pathway	Associated genera
Alkane 1‐monooxygenase (*alkB*)	*Alcanivorax*, *Burkholderia*, *Marinobacter*, *Pseudomonas*
Acyl Co‐A dehydrogenase (*fadE*)	*Chromohalobacter*, *Marinobacter*, *Pseudoalteromonas*, *Shewanella*
Lipases	*Aeromonas*, *Burkholderia*, *Colwellia*, *Rhodopirellula*, *Pseudomonas*, *Shewanella*
Oxidative phosphorylation	*Alcanivorax*, *Chromohalobacter*, *Colwellia*, *Flavobacterium*, *Marinobacter*, *Pseudomonas*, *Shewanella*
Nitrogen cycling	*Alcanivorax*, *Alteromonas*, *Colwellia*, *Marinobacter*, *Marinomonas*, *Pseudoalteromonas*, *Pseudomonas*, *Psychromonas*
Sulphur cycling	*Colwellia*, *Marinobacter*, *Marinomonas*, *Pseudoalteromonas*, *Pseudomonas*, *Saccharophagus*, *Shewanella*

Functional gene expression was dominated by gammaproteobacteria and included the orders Oceanospirillales, Pseudomonadales, and Alteromonadales (Figure S3). Genera within the Oceanospirillales and Pseudomonadales were both enriched by the presence of oil, Corexit, or both at 5 days, including *Alcanivorax*, *Bermanella*, *Marinomonas*, and *Pseudomonas*. Some Alteromonadales genera were enriched by the presence of either oil or Corexit (*Alteromonas*, *Marinobacter*, *Saccarophagus*, *Shewanella*), however, some genera were only enriched by the presence of Corexit (*Colwellia*, *Psychromonas*, *Moritella*). There was no indication that the addition of Corexit had a negative impact on the presence or abundance of sequences affiliated with genera enriched in the presence of oil alone, which has previously been reported with 16S rRNA gene sequences for the oil‐degrading taxa *Marinobacter* and *Cytoclasticus* in Gulf of Mexico seawater mesocosm incubations (Kleindienst et al., 2015).

## DISCUSSION

The bacterial community present in Arctic seawater responded to the introduction of crude oil, Corexit 9500, or both, by upregulating the expression of significantly different suites of genes. The constellation of genes upregulated differed significantly overall based on the mixtures added, with some genes being upregulated more in response to either Corexit or crude oil. The differences in the metatranscriptomes of the different treatments were largely explained by the expression of energy‐metabolism gene classes, which include those associated with oxidative phosphorylation, photosynthesis, nitrogen metabolism, and sulphur metabolism, all of which correlated with overall metatranscriptomic structure (Figure 1, Table S1).

The enrichment of bacterial and *rpoB* transcripts in the presence of Corexit suggests that Corexit may enhance bacterial activity, even when it is co‐present with oil (Figures S1 and S2). The stimulation of bacterial communities by Corexit alone or with oil has been previously observed in other seawater incubation studies using cell counts (Hazen et al., 2010; Kleindienst et al., 2015; Lindstrom & Braddock, 2002) and qPCR of 16S rRNA genes (McFarlin et al., 2018), which all increased in the presence of Corexit relative to controls. In this experiment, the relative abundance of *rpoB* transcripts returned to basal levels between 5 and 30 days, although the temporal resolution of our study design could not detect interim times; also, responses may differ for an in situ spill where different conditions may prevail. We observed that early in the incubation, fatty acid gene expression in the oil‐only treatment was dominated by *alkB*, which catalyses alkane degradation, whereas Corexit‐only and oil + Corexit treatments were dominated by *fadE*, which catalyses the initial step of β‐oxidation of fatty acids. (Figure 3). The oil + Corexit treatment appeared to be an amalgamation of the oil‐only and Corexit‐only treatments, with the expression of *alkB* and *fadE* genes falling between the two groups. Additionally, the subsequent β‐oxidation step enzymes *fadA* and *fadB* were enriched in the presence of Corexit. Previous functional gene analyses, both in situ in contaminated water following the Deepwater Horizon spill and from incubation studies, also found that the presence (Lu et al., 2012) or expression (Doyle et al., 2020; Mason et al., 2012; Tremblay et al., 2019) of alkane degradation genes such as *alkB* were increased, although it is unknown which genes were associated with oil versus Corexit. However, in one study, an increased intensity and richness of *alkB* genes was observed using a GeoChip microarray assay of Arctic seawater exposed to either oil or Corexit alone relative to controls, indicating that this gene or pathway may be involved in the degradation of both (McFarlin et al., 2018). This is further supported by recent evidence that proteins involved in alkane and non‐linear hydrocarbon metabolism were upregulated in a *Marinobacter* species in the presence of Corexit relative to oil‐only and control treatments (Rughöft et al., 2021).

**FIGURE 5:**
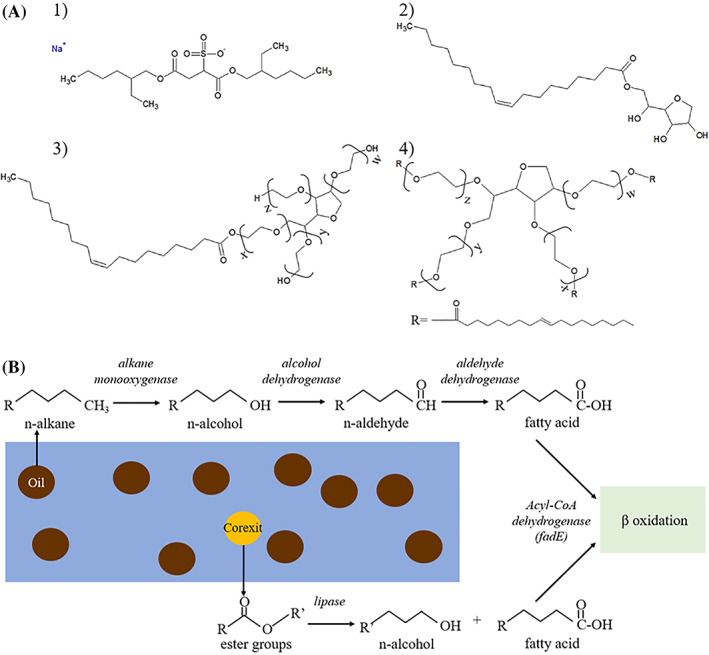
(A) Chemical structures of the Corexit 9500 components (1) dioctyl sodium sulfosuccinate (DOSS), (2) Span 80 (sorbitan monooleate, (3) Tween 80 (sorbitan monooleate polyethoxylated), and (4) Tween 85 (sorbitan trioleate polyethoxylate). (B) Proposed bacterial biodegradation pathway for the major Corexit 9500 surfactant components and comparison to petroleum hydrocarbon alkane degradation based on metatranscriptomic data from Arctic seawater mesocosms amended with Corexit and/or oil or no treatment (control)

The presence or expression of β‐oxidation genes such as *fadE*, *fadA*, and *fadB* have been observed in waters exposed to dispersed oil, both in field (Rivers et al., 2013) and incubation studies (Ribicic et al., 2018), but it remains unclear whether those findings are associated with the oil, dispersant, or both. Recent incubation studies have also demonstrated an upregulation of β‐oxidation gene transcripts in treatments that contained Corexit compared to oil‐only treatments and unamended controls (Doyle et al., 2020; Peña‐Montenegro et al., 2020).Based on the significant enrichment of *fadE*, *fadA*, and *fadB* genes at the 5‐day time point, increased β‐oxidation of chemical compounds was likely occurring in the presence of Corexit and oil + Corexit relative to oil‐only or the control. This agrees with previously reported chemical loss data from this experiment, which showed that the majority of Corexit surfactant loss occurred within 5 days and the majority of oil loss within 10 days (Figure 3; Gofstein et al., 2020). When examining the constituents of Corexit 9500 compared to crude oil, their chemical structures, which contain multiple ester groups, (Figure 5A) have functional groups that may lead to their being more labile than the alkanes present in crude oil due to fewer steps being required to prepare esters to undergo β‐oxidation (Figure 5B).

Based on these findings, we propose a hypothetical pathway for the biodegradation of the major surfactant components in Corexit (Figure 5B). The pathway involves the transformation of Corexit surfactant esters into fatty acids, which can then undergo β‐oxidation beginning with *fadE*. Plausible mechanisms to prepare esters for β‐oxidation, which metabolizes fatty acid substrates, may include processes such as hydrolysis with an esterase (Plou et al., 1998; Rojo, 2009) or abiotically (Lundberg & Stjerndahl, 2011). While significant upregulation of esterase transcripts in the presence of Corexit was not observed in this study, it is possible that the temporal resolution of the samples used may not have captured this part of the proposed pathway. The alcohols produced from the ester hydrolysis can also undergo further oxidation to fatty acids to enter the β‐oxidation pathway (Figure 5B). Compared to the degradation of alkanes, which must undergo several oxidation steps to be transformed into fatty acids prior to undergoing β‐oxidation, this proposed pathway for Corexit compounds funnels directly into central metabolic pathways that are widespread in prokaryotes. The general prevalence of these enzymes in the environment may serve to explain the rapid degradation of some Corexit components and the variety of organisms observed in response to Corexit (Gofstein et al., 2020; Kleindienst et al., 2015; McFarlin et al., 2018; Techtmann et al., 2017). Based on our previously reported chemical loss data for Corexit components (Gofstein et al., 2020), which showed rapid degradation of the non‐ionic surfactants (Span 80 and Tweens 80 + 85) within 5 days, the community shifts and functional genes observed at 5 days may be associated with degradation of the remaining Corexit components, such as DOSS (Figure 3). However, the non‐ionic components of Corexit 9500 and DOSS both contain ester functional groups that can potentially allow them to enter the proposed degradation pathway described here (Figure 5). The enrichment of transcripts related to several of the biodegradation processes observed here in response to oil and/or Corexit was associated with multiple taxa (Table 1), which has been previously reported in other studies (Brakstad et al., 2015; Dombrowski et al., 2016; Doyle et al., 2020; Handley et al., 2017). The *alkB* and *fadE* genes expressed originated from different genera, except for *Marinobacter*, which expressed both. Broader metabolic functions were otherwise generally associated with the same groups of genera regardless of treatment (Table 1). This functional redundancy suggests that multiple taxa may be capable of contributing to the degradation of Corexit components, either completely or in part as members of a diverse microbial consortium.

These findings provide new insight into the potential genes, pathways, and microbial consortia involved in the biodegradation of Corexit 9500 in the Arctic marine environment. While the time points available for analysis here were limited by the complexity and size of the original incubation study, this lays the groundwork for future studies, especially those focusing on earlier stages of Corexit biodegradation. Further research, such as using microbial culture and/or stable isotope probing (SIP) of Corexit components, would help to confirm the use of the β‐oxidation pathway in Corexit surfactant degradation and elucidate which taxa are directly involved in its degradation. Doing so will bolster our understanding of the fate of Corexit 9500 and related chemical dispersants in the Arctic – and global – marine environments.

## CONFLICT OF INTEREST

The authors declare no conflicts of interest.

## Supporting information


**Appendix S1** Supporting information

## Data Availability

The metatranscriptomics sequences used in this study are available on the MG‐RAST metagenomics analysis server under project mgp88578.
