# Supplementary material for: Metatranscriptomic shifts suggest shared biodegradation pathways for Corexit 9500 components and crude oil in Arctic seawater
Source: Environ Microbiol Rep. 2022 Sep 29;15(1):51–9. doi: 10.1111/1758-2229.13127 (PMC10103760; doi:10.1111/1758-2229.13127)
Supplement: Supplementary file 1 — Appendix S1 Supporting information [file EMI4-15-51-s001.docx]

**Supporting Information**

**Results and Discussion**

*Nitrogen and Sulfur Cycling Gene Expression*

Nitrogen-cycling gene expression in all experimental treatments consisted primarily of those encoding enzymes required for ammonia assimilation including glutamate dehydrogenase (*gdh*) and glutamate synthase (*GOGAT*), which is consistent with the rapid decrease in seawater ammonia concentrations in these experimental treatments beginning at 5 days (Gofstein *et al.*, 2020). The expression of nitrate reductase (*napA*) and nitrite reductase (*nirB*) genes was also detected, indicating that assimilatory nitrate reduction was also occurring. The enrichment of sulfur-processing genes in treatments containing Corexit included adenylylsulfate kinase (*cysC*), sulfate adenylyltransferase (*cysD,* *cysN*), phosphoadenosine phosphosulfate reductase (*cysH*), and sulfite reductase (*cysJ*), indicating that assimilatory sulfate reduction was occurring.

The increased transcription of assimilatory nitrogen-cycling genes in all of the experimental treatments would help to support the increased bacterial metabolism previously observed here and in other oil biodegradation experiments (Lindstrom and Braddock, 2002; Hazen *et al.*, 2010; McFarlin *et al.*, 2014, 2018; Kleindienst *et al.*, 2015). Increases in nitrogen-related metabolic processes have also been observed with molecular methods in post-spill Deepwater Horizon water and sediments, including ammonia assimilation (Scott *et al.*, 2014), assimilatory nitrate reduction (Lu *et al.*, 2012), denitrification (Mason *et al.*, 2014; Scott *et al.*, 2014; Handley *et al.*, 2017), and dissimilatory nitrate reduction (Rivers *et al.*, 2013; Scott *et al.*, 2014), suggesting that multiple pathways are utilized to support the high nitrogen requirement for oil and/or Corexit biodegradation, depending on the available nitrogen resources. This has been further corroborated by incubation studies, where the presence or expression of genes encoding for assimilatory nitrate reduction (Nanjappa *et al.*, 2021), dissimilatory nitrate reduction (Nanjappa *et al.*, 2021), and N_2_ fixation (Sun *et al.*, 2019; Peña-Montenegro *et al.*, 2020) were shown to be upregulated in the presence of Corexit.

The enrichment of assimilatory sulfate reduction transcripts in the presence of Corexit (Fig. 2) suggests that microorganisms may utilize the sulfate group from the degradation of DOSS, although the resolution of our chemical analyses are unable to corroborate this. Upregulation of assimilatory sulfate reduction transcripts by *Colwellia* and *Marinobacter* has also been previously observed in seawater incubations containing Corexit (Peña-Montenegro *et al.*, 2020). Increased sulfur cycling (Hazen *et al.*, 2010) and uptake (Mason *et al.*, 2012), including assimilatory (Lu *et al.*, 2012) or generalized (Dombrowski *et al.*, 2016) sulfate reduction, was also observed in Deepwater Horizon dispersed oil plume samples. This may be attributable not only to the increased nutrient requirements associated with elevated microbial activity during biodegradation, but also may be in part to the degradation of sulfonated compounds present in oil and/or DOSS.

**References**

Dombrowski, N., Donaho, J.A., Gutierrez, T., Seitz, K.W., Teske, A.P., and Baker, B.J. (2016) Reconstructing metabolic pathways of hydrocarbon-degrading bacteria from the Deepwater Horizon oil spill. *Nat Microbiol* **1**: 1–7.

Gofstein, T.R., Perkins, M., Field, J., and Leigh, M.B. (2020) The Interactive Effects of Crude Oil and Corexit 9500 on Their Biodegradation in Arctic Seawater. *Appl Environ Microbiol* **86**:.

Handley, K.M., Piceno, Y.M., Hu, P., Tom, L.M., Mason, O.U., Andersen, G.L., et al. (2017) Metabolic and spatio-taxonomic response of uncultivated seafloor bacteria following the Deepwater Horizon oil spill. *ISME J* **11**: 2569–2583.

Hazen, T.C., Dubinsky, E. a, DeSantis, T.Z., Andersen, G.L., Piceno, Y.M., Singh, N., et al. (2010) Deep-sea oil plume enriches indigenous oil-degrading bacteria. *Science (80- )* **330**: 204–208.

Kleindienst, S., Seidel, M., Ziervogel, K., Grim, S., Loftis, K., Harrison, S., et al. (2015) Chemical dispersants can suppress the activity of natural oil-degrading microorganisms. *Proc Natl Acad Sci* **112**: 14900–14905.

Lindstrom, J.E. and Braddock, J.F. (2002) Biodegradation of petroleum hydrocarbons at low temperature in the presence of the dispersant Corexit 9500. *Mar Pollut Bull* **44**: 739–747.

Lu, Z., Deng, Y., Van Nostrand, J.D., He, Z., Voordeckers, J., Zhou, A., et al. (2012) Microbial gene functions enriched in the Deepwater Horizon deep-sea oil plume. *ISME J* **6**: 451–460.

Mason, O.U., Han, J., Woyke, T., and Jansson, J.K. (2014) Single-cell genomics reveals features of a Colwellia species that was dominant during the Deepwater Horizon oil spill. *Front Microbiol* **5**: 1–8.

Mason, O.U., Hazen, T.C., Borglin, S., Chain, P.S.G., Dubinsky, E.A., Fortney, J.L., et al. (2012) Metagenome, metatranscriptome and single-cell sequencing reveal microbial response to Deepwater Horizon oil spill. *ISME J* **6**: 1715–1727.

McFarlin, K.M., Perkins, M.J., Field, J.A., and Leigh, M.B. (2018) Biodegradation of Crude Oil and Corexit 9500 in Arctic Seawater. *Front Microbiol* **9**: 1–14.

McFarlin, K.M., Prince, R.C., Perkins, R., and Leigh, M.B. (2014) Biodegradation of dispersed oil in Arctic seawater at -1°C. *PLoS One* **9**: 1–8.

Nanjappa, D., Liang, Y., Bretherton, L., Brown, C., Quigg, A., Irwin, A.J., and Finkel, Z. V. (2021) Contrasting transcriptomic responses of a microbial eukaryotic community to oil and dispersant. *Environ Pollut* **288**: 117774.

Peña-Montenegro, T.D., Kleindienst, S., Allen, A.E., Eren, A.M., McCrow, J.P., Sánchez-Calderón, J.D., et al. (2020) Colwellia and Marinobacter metapangenomes reveal species-specific responses to oil and dispersant exposure in deepsea microbial communities. *bioRxiv* 2020.09.28.317438.

Rivers, A.R., Sharma, S., Tringe, S.G., Martin, J., Joye, S.B., and Moran, M.A. (2013) Transcriptional response of bathypelagic marine bacterioplankton to the Deepwater Horizon oil spill. *ISME J* **7**: 2315–2329.

Scott, N.M., Hess, M., Bouskill, N.J., Mason, O.U., Jansson, J.K., and Gilbert, J.A. (2014) The microbial nitrogen cycling potential is impacted by polyaromatic hydrocarbon pollution of marine sediments. *Front Microbiol* **5**: 1–8.

Sun, X., Chu, L., Mercando, E., Romero, I., Hollander, D., and Kostka, J.E. (2019) Dispersant enhances hydrocarbon degradation and alters the structure of metabolically active microbial communities in shallow seawater from the northeastern gulf of mexico. *Front Microbiol* **10**: 1–15.

**Figures**


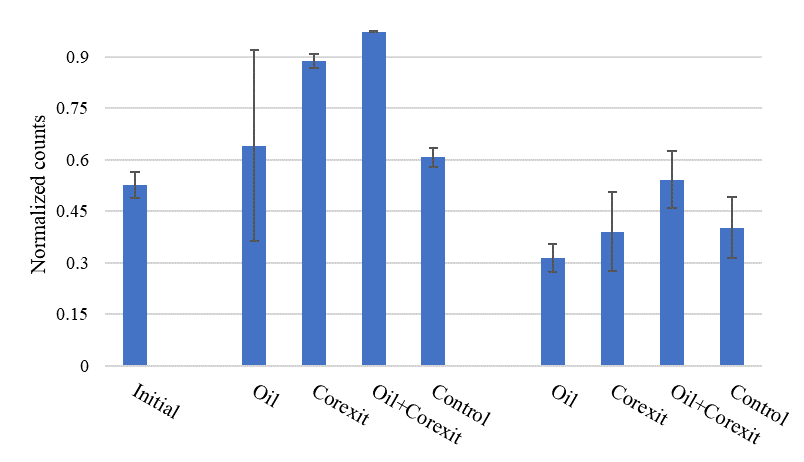


Day 5

Day 30

Day 0

Figure S1. Normalized transcript counts originating from bacteria at 0, 5, and 30 days in Arctic seawater mesocosms amended with Corexit and/or oil or no treatment (control). The error bars represent one standard deviation from the mean.


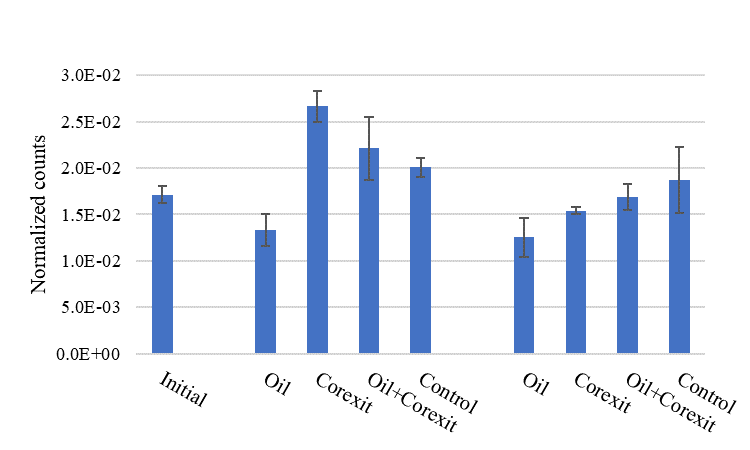


Day 0

Day 5

Day 30

Figure S2. Normalized counts of RNA polymerase B (*rpoB*) at 0, 5, and 30 days in Arctic seawater mesocosms amended with Corexit and/or oil or no treatment (control). The error bars represent one standard deviation from the mean.


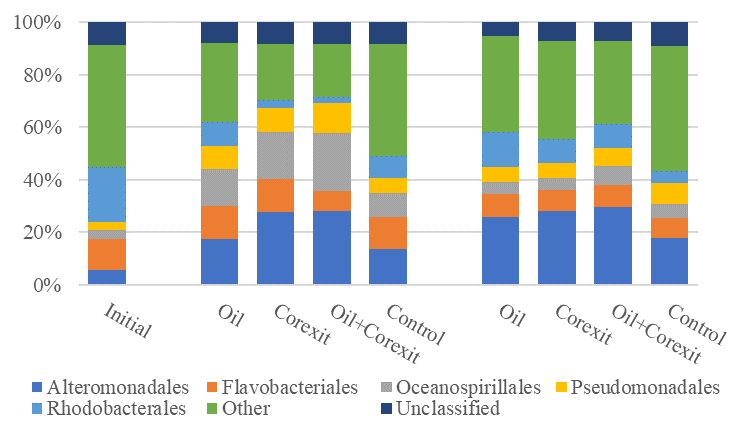


Day 5

Day 30

Day 0

Figure S3. Relative abundance of the bacterial orders associated with metabolic gene expression in Arctic seawater mesocosms amended with Corexit and/or oil or no treatment (control) at 0, 5, and 30 days incubation.

Table S1. Mantel test statistics and associated p-values for the vector analysis of specific KEGG metabolic gene classes to the overall metatranscriptomic profile in Arctic seawater mesocosms amended with Corexit and/or oil or no treatment (control). P-values <0.05 are denoted with an asterisk (*).

|  | **Mantel test statistic, r** | **p-value** |
| --- | --- | --- |
| Time | 0.34 | 0.0010 * |
| Lipids | 0.34 | 0.0010 * |
| Fatty acids | 0.37 | 0.0020 * |
| *alkB* | 0.14 | 0.0901 |
| *fadE* | 0.55 | 0.0010 * |
| Glycerolipids | 0.33 | 0.0010 * |
| Glycerophospholipids | 0.32 | 0.0010 * |
| Energy metabolism | 0.74 | 0.0010 * |
| Oxidative phosphorylation | 0.59 | 0.0010 * |
| Photosynthesis | 0.76 | 0.0010 * |
| Nitrogen | 0.48 | 0.0010 * |
| Sulfur | 0.09 | 0.1832 |
| Xenobiotics | 0.05 | 0.2893 |
